# Intrinsic Disorder of the C-Terminal Domain of *Drosophila* Methoprene-Tolerant Protein

**DOI:** 10.1371/journal.pone.0162950

**Published:** 2016-09-22

**Authors:** Marta Kolonko, Katarzyna Ożga, Rafał Hołubowicz, Michał Taube, Maciej Kozak, Andrzej Ożyhar, Beata Greb-Markiewicz

**Affiliations:** 1 Department of Biochemistry, Faculty of Chemistry, Wrocław University of Science and Technology, Wybrzeże Wyspiańskiego 27, 50–370, Wrocław, Poland; 2 Joint Laboratory for SAXS studies, Faculty of Physics, Adam Mickiewicz University, Umultowska 85, 61–614, Poznań, Poland; 3 Department of Macromolecular Physics, Faculty of Physics, Adam Mickiewicz University, Umultowska 85, 61–614, Poznań, Poland; Danish Cancer Society Research Center, DENMARK

## Abstract

Methoprene tolerant protein (Met) has recently been confirmed as the long-sought juvenile hormone (JH) receptor. This protein plays a significant role in the cross-talk of the 20-hydroxyecdysone (20E) and JH signalling pathways, which are important for control of insect development and maturation. Met belongs to the basic helix-loop-helix/Per-Arnt-Sim (bHLH-PAS) family of transcription factors. In these proteins, bHLH domains are typically responsible for DNA binding and dimerization, whereas the PAS domains are crucial for the choice of dimerization partner and the specificity of target gene activation. The C-terminal region is usually responsible for the regulation of protein complex activity. The sequence of the Met C-terminal region (MetC) is not homologous to any sequence deposited in the Protein Data Bank (PDB) and has not been structurally characterized to date. In this study, we show that the MetC exhibits properties typical for an intrinsically disordered protein (IDP). The final averaged structure obtained with small angle X-ray scattering (SAXS) experiments indicates that intrinsically disordered MetC exists in an extended conformation. This extended shape and the long unfolded regions characterise proteins with high flexibility and dynamics. Therefore, we suggest that the multiplicity of conformations adopted by the disordered MetC is crucial for its activity as a biological switch modulating the cross-talk of different signalling pathways in insects.

## Introduction

Insect development is regulated by the coordination of two hormone-signalling pathways: 20-hydroxyecdysone (20E) and juvenile hormone (JH) [1]. Although the 20E receptor has been studied extensively and its mechanism of action is described in detail, the identity and function of the JH receptor has long remained elusive [2]. Recent studies of a protein known as Methoprene tolerant protein (Met) have suggested that Met acts as the JH receptor [3,4]. Met binds JH [3,5] and is important for the function of JH in preventing the precocious development of adult structures during metamorphosis from larva to pupa [6]. In *Drosophila*, additionally to Met, exists its paralog known as germ cell-expressed protein (Gce). Gce is capable of rescuing Met function in *Met* null mutants and ensuring their survival [7]. Importantly, the functions of Met and Gce are not fully redundant [8].

Met and Gce belong to a family of basic helix-loop-helix/Per-Arnt-Sim (bHLH-PAS) transcription factors [9,10]. bHLH-PAS family members are responsible for the regulation of important developmental and physiological processes [11,12] and present a relatively well-conserved domain structure [13]. bHLH domains are typically responsible for DNA binding and dimerization, whereas PAS domains are crucial for selecting dimerization partner and ensuring the specificity of target gene activation [14]. The C-terminal fragment of bHLH-PAS transcription factors is usually responsible for the regulation of protein complex activity [11,12]. Such regulation takes place in the mammalian proteins SIM1 (single minded 1) and SIM2 (single minded 2), both of which are bHLH-PAS transcription factors that regulate the expression of genes in midline cells during development. SIM1 and SIM2 exhibit highly homologous PAS domains (90% of sequence similarity), but they are highly divergent at their C-termini [13]. Both proteins have been shown to heterodimerize with ARNT (aryl hydrocarbon nuclear translocator). However, because of differences in their C-terminal fragments, the heterodimers exhibit opposing activities: the SIM1:ARNT heterodimer activates and SIM2:ARNT represses target gene expression. This functional divergence is attributed to the C-terminus of SIM2, which contains two repressive domains that suppress the transactivation domain (TAD) of ARNT [15].

High sequence homology between Met and Gce is observed for the bHLH (78%), PAS-A (68%) and PAS-B (86%) domains [10] but not between the C-termini of Met and Gce. These regions present features of a TAD and include regions rich in glutamine (Q_R_) and aspartic acid (D_R_) [16]. Bernardo and Dubrovsky [17] have shown that the C-termini of Met and Gce are responsible for their interactions with orphan nuclear receptor Fushi Tarazu factor-1 (FTZ-F1), a nuclear receptor (NR) that has been shown to bind the *Drosophila* Fushi Tarazu protein (Ftz) and influence its DNA binding specificity [18]. Interestingly, the modes of interaction with FTZ-F1 differ for Met and Gce. Both proteins utilize a conserved novel NR box (LIXXLL sequence), but only Met uses an Q_R_ region as a secondary interaction site [17]. Furthermore, the C-terminal regions of both Met and Gce contain a nuclear export signal (NES) [19,20], but only Gce contains a dominant nuclear localization signal (NLS) [20]. Together, these data suggest the importance of the C-termini in functional differentiation (i.e., the regulation of the transcription of distinct sets of genes by these proteins) between Met and Gce [16].

To date, no information about the structure of the Met C-terminus (MetC) has been published. Therefore, we developed and optimized a protocol for the efficient expression and purification of MetC to obtain a homogenous preparate for comprehensive *in vitro* structural analysis and *in silico* examination. All of our results indicate that MetC exhibits properties typical for an intrinsically disordered protein (IDP), and has no propensity to create oligomers in solution. It was long believed that a stable three-dimensional (3-D) protein structure is necessary for its function. However, recent research has demonstrated that many proteins lack a folded structure under physiological conditions [21] but nevertheless perform defined functions [22]. Such proteins are referred to as “intrinsically disordered proteins” (IDPs) [23,24]. Moreover, proteins with essentially stable tertiary structures (i.e., globular proteins) often contain shorter disordered fragments (intrinsically disordered regions [IDRs]) and perform defined functions. Interestingly, a significant portion of transcription factors exhibit properties of IDRs [25,26]. Unfolding studies and small-angle X-ray scattering (SAXS) experiments revealed that MetC is not fully disordered, what could be crucial in protein recognition. Therefore, we suggest that these structured fragments could facilitate binding to partners via disorder-to-order transitions. The possible influence of the structural properties of MetC and the role of its flexibility in *Drosophila* development are discussed in this article.

## Materials and Methods

### *In silico* analysis

Disorder predictions were made using the IUPred server [27,28] available at http://iupred.enzim.hu. Amino acid composition analyses were performed using the Composition Profiler available at http://www.cprofiler.org [24,29]. The Uversky plot [30], VSL2 [31,32], PONDR-FIT [33], DISOPRED2 [34] and FoldIndex [35] calculations were made using PONDR at http://www.pondr.com. GeneSilico MetaDisorder server [36] was used for results averaging. All analyses were performed using the default settings.

### Chemicals

All buffers were prepared at room temperature and titrated to pH 7.5. Buffer A was 20 mM Na_2_HPO_4_ and 100 mM NaCl. Buffer B was 50 mM Na_2_HPO_4_ and 350 mM NaCl. Buffer C was 50 mM Na_2_HPO_4_, 350 mM NaCl and 200 mM imidazole. Buffer D was 20 mM Tris-HCl. Buffer E was 20 mM Tris-HCl and 1 M NaCl. Buffer F was 20 mM Tris-HCl and 150 mM NaCl.

### Expression vector preparation

cDNA encoding full-length *D*. *melanogaster* Met protein was kindly provided by Prof. Thomas G. Wilson (Ohio State University) and used as a polymerase chain reaction (PCR) template. The *E*. *coli* strain XL1-Blue (Invitrogen) was used as the host strain during cloning. A fragment of cDNA corresponding to the C-terminus of Met (627 bp, aa residues 509–716) was amplified using two oligonucleotides as primers. The forward primer was 5’gcgccatatgGCGGGCCGGCAAAAGGTG3’, and the reverse primer was 5’gcgcgaattcTCATCGCAGCGTGCTGGTC3’. The primers introduced restriction sites for *Nde*I and *Eco*RI, respectively (underlined). The lower-case letters in the primer sequences correspond to nucleotides added to the coding sequences during the cloning procedure, whereas the upper case letters represent sequence present in *MetC* cDNA. The resulting fragment was double-digested with *Nde*I and *Eco*RI and cloned into the pColdTF^TM^ DNA vector (Takara). The fragment was inserted in frame with the translation-enhancing element (TEE), a hexahistidine tag (6×His tag), and a Trigger factor protein (TF). The presence of the insert in pCold vector was confirmed by a restriction analysis, and the final construct pCold/TEE-6 x His-TF-MetC was verified by DNA sequencing.

### Expression and purification of MetC

The BL21(DE3)pLysS *E*.*coli* strain (Novagen) was transformed with 2 ng of purified recombinant pCold^TM^ TF DNA vector (Takara) encoding TEE-6 x His-TF-MetC and was plated on Lysogeny Broth (LB) agar containing 35 μg/ml chloramphenicol and 50 μg/ml carbenicillin. After 16 h of incubation at 37°C, a single colony was used to inoculate 20 ml of LB medium containing appropriate amounts of antibiotics (35 μg/ml chloramphenicol and 50 μg/ml carbenicillin). The culture was incubated overnight in a rotary shaker operated at 182 rpm at 29°C. Ten millilitres of starting culture were used to inoculate 300 ml of Terrific Broth (TB) medium with antibiotics. The incubation was conducted under the same conditions until the optical density (OD_600_) reached 0.6–0.7. Subsequently, the culture was cooled to 15°C and incubated for 30 min. Expression of the recombinant protein was induced by the addition of 0.25 mM isopropyl-β-thiogalactopyranoside (IPTG). After 20 h of incubation at 15°C, the cells were harvested by centrifugation at 4000 × g (20 min, 4°C), resuspended in 10 ml of buffer A (20 mM Na_2_HPO_4_ and 100 mM NaCl) supplemented with 0.2 mg/ml phenylmethylsulfonyl fluoride (PMSF) and frozen at -80°C.

The frozen cells were disrupted by subsequent thawing and freezing [37]. The cell suspension was supplemented with 0.2 mg/ml PMSF and 1 mM β-mercaptoethanol (βME). DNase I (20 μg/ml) and RNase A (20 μg/ml) were added to facilitate the complete digestion of the nucleic acids. Next, the cell extract was centrifuged at 18000 × g for 1 h at 4°C to obtain a soluble fraction containing TEE-6×His-TF-MetC. The expressed recombinant protein had a 6×His tag, so immobilized metal affinity chromatography (IMAC) has been chosen as the first step of purification. We used Co^2+^-TALON resin (Clontech). The binding and elution conditions recommended by the manufacturer (Clontech Laboratories, Inc.) were applied. The soluble fractions obtained after cell lysis were incubated and agitated for 1 h at 4°C with 2 ml of Co^2+^-TALON resin that had been equilibrated with buffer B (50 mM Na_2_HPO_4_ and 350 mM NaCl). The resin was loaded on a reusable column (20 ml, Clontech) and washed with 20 ml of buffer B. The fusion protein was eluted with 10 ml of buffer C (50 mM Na_2_HPO_4_, 350 mM NaCl, and 200 mM imidazole). One-millilitre fractions were collected, and the protein concentration (A_280_) was measured. Selected protein fractions containing the fusion protein were combined, and the buffer was changed to buffer D (20 mM Tris-HCl) on the Amicon Ultra Centrifugal Filter (Milipore) with a cut-off of 10 kDa. Next, TEE-6×His-TF-MetC was digested with thrombin (Merck Millipore). The digestion of construct expressed from pColdTF^TM^ vector leaves a flexible tail (GSGGIEGRH) upstream of the MetC sequence (AGR…). The digestion conditions were optimized, and 5 U of thrombin was used to digest 1 mg of fusion protein at 20°C over 2 h. The digestion products were concentrated to 2 ml by ultrafiltration on the Amicon Ultra Centrifugal Filter (Milipore) with a cut-off of 10 kDa. The next step of MetC purification exploited the characteristics of MetC and the TEE-6×His-TF tag. Because the calculated pI of MetC (6.25) differs from that of TEE-6×His-TF tag (5.02), anion exchange chromatography was used. The MonoQ column (Amersham Pharmacia Biotech) was connected to an ÄKTAexplorer (Amersham Biosciences) system operated at 0.5 ml/min at room temperature. Detection was performed by monitoring the ultraviolet (UV) absorbance at 220 and 280 nm. The concentrated digestion products were passed over the column. The MonoQ column was washed with buffer D (20 mM Tris-HCl) for 5 min, and the proteins were eluted in a linear gradient of NaCl (0–0.5 M) for 30 min (buffer E, 20 mM Tris-HCl and 1 M NaCl). Finally, the collected MetC protein was dialysed three times against buffer F (20 mM Tris-HCl and 150 mM NaCl) at 4°C and with mixing.

### Sodium dodecyl sulfate polyacrylamide gel electrophoresis (SDS-PAGE)

Samples from the expression and purification steps were analysed by SDS-PAGE using 12% polyacrylamide gels developed in a Tris/glycine system [38]. Unstained Protein Molecular Weight Marker (PMWM) (MBI Fermentas) was used as a molecular mass protein standard. After SDS-PAGE analysis, the gels were stained with Coomasie Brilliant Blue R-250 [39].

### Determination of protein concentration

The concentration of purified protein was measured spectrophotometrically at 280 nm. The absorption coefficient of MetC (0.130 dm^3^·g^−1^·cm^−1^) was calculated using the ProtParam tool [40], which is available at http://us.expasy.org/tools/protparam.html.

### Electrospray ionization (ESI) mass spectrometry

Ten micrograms of purified MetC were desalted by reverse-phase chromatography. PepRPC HR 5/5 column (Amersham Bioscience) was connected to the ÄKTAexplorer (Amersham Biosciences) system and equilibrated with 0.05% trifluoroacetic acid (TFA). A linear gradient of acetonitrile (0–80%) at a flow rate of 1 ml/min was applied for 25 min. The eluted protein was used in the next step of experiment. High-resolution mass spectrometry was performed using a micrOTOF-Q^TM^ spectrometer (Bruker Daltonics) equipped with an Apollo II ESI source and an ion funnel. The mass spectrometer was operated in positive ion mode at 180°C. The solution flow rate was 3 μl/min. The instrument was calibrated with a Tunemix mixture (Bruker Daltonik) in the quadratic regression mode. Data acquisition was achieved using micrOTOFcontrol 2.0 software (Bruker Daltonics). The mass resolution was 1500 full width at half maximum (FWHM). Data analysis was performed using DataAnalysis^TM^ software (Bruker Daltonics). The theoretical molecular mass value was calculated using the ProtParam tool, which is available at http://us.expasy.org/tools/protparam.html.

### Circular dichroism (CD) spectroscopy

CD spectra were recorded using a JASCO J-815 CD-spectropolarimeter with the sample cell temperature control unit (Peltier Type Control System). All measurements were collected at 20°C in a 2 mm path-length cuvette 100QS (Hellma). The spectra consisted of an average of five scans at a speed of 20 nm/min, with a data resolution of 1.0 nm and a 1.0 nm bandwidth in the spectral range of 190–260 nm. The MetC concentration was 18 μM. Non-denatured protein was examined in buffer F (20 mM Tris-HCl and 150 mM NaCl). Additional measurements were performed after a 1 h incubation in the same buffer supplemented with appropriate amounts of guanidine hydrochloride (GdmCl), 2,2,2-trifluoroethanol (TFE), or trimethylamine N-oxide (TMAO). All results, with acceptable high tension (HT under 750 V, S1 Fig), were converted to molar residual ellipticity units (all data presented in S1 Table). CD spectra were analysed using CDPro spectra software [41]. The CONTINLL algorithm on the SDP48 base [41] was used for CD spectrum deconvolution.

### Size-exclusion chromatography (SEC)

A Superdex75 10/300 GL (Amersham Pharmacia Biotech) column was connected to the ÄKTAexplorer (Amersham Biosciences) system and operated at 0.5 ml/min at room temperature. Detection was performed by monitoring the UV absorbance at 220 and 280 nm. The column was equilibrated with buffer F (20 mM Tris-HCl and 150 mM NaCl) and calibrated using the following standard proteins: apoferritin (443 kDa, 64.8 Å), β-amylase (200 kDa, 48.8 Å), alcohol dehydrogenase (150 kDa, 44.0 Å), albumin (66 kDa, 32.9 Å), and carbonic anhydrase (29 kDa, 24.5 Å). All Stokes radii (R_S_) of the standard proteins were calculated according to Eq 1 [42]:

$$\log\left(R_{S}\right)=-(0.204\pm 0.023)+(0.357\pm 0.005)\log\left(MW\right)$$ (1)

Purified MetC (1 mg/ml) was injected in a volume of 0.1 ml. The total column volume (V_T_) was 24 ml, and the column void volume (V_0_), as determined using blue dextran, was 8.18 ± 0.11 ml. The elution volume (V_E_) of each standard protein was used to calculate the gel-phase distribution coefficients (K_AV_ factors) according to Eq 2 [43]. K_AV_ values were plotted against the calculated R_S_ and fitted to the standard curve. Finally, the R_S_ of MetC was calculated.

$$K_{AV}=\frac{V_{E}-V_{0}}{V_{T}-V_{0}}$$ (2)

### Analytical ultracentrifugation (AUC)

Sedimentation velocity experiments were conducted using a Beckman Coulter ProteomeLab XL-I ultracentrifuge (Beckman Coulter Inc.). An An-60Ti rotor and cells equipped with sector-shaped two-channel charcoal-filled Epon centrepieces were used. Sample sectors were filled with 400 μl of MetC at three concentrations: 0.08, 0.17 and 0.30 mg/ml. All measurements were performed in buffer F (20 mM Tris-HCl and 150 mM NaCl). The experiment was conducted overnight in 20°C at 50000 rpm, and the absorbance scans were collected at 230 nm. Time-corrected scans representing the whole sedimentation process were analysed using SEDFIT, which is available at http://www.analyticalultracentrifugation.com [44]. The partial specific volumes of proteins and the density and dynamic viscosity of the buffer at 20°C were calculated using the SEDNTERP software available at http://sednterp.unh.edu/ [45]. A sedimentation coefficient distribution [(c(s)] model was used to calculate the sedimentation coefficients (s) and the frictional ratios (f/f_0_) from the acquired data. In addition, water-standardized sedimentation coefficients (s_20,w_), R_S_, and apparent molecular mass (MMs) were calculated.

### Small angle X-ray scattering (SAXS)

SAXS experiments were performed on beamline P12 of the EMBL Hamburg Oustation at the Petra III storage ring at the Deutsches Elektronen-Synchrotron (DESY) in Hamburg [46]. Thirty-microlitre samples of MetC at a concentration of 2.6 mg/ml and buffer F (20 mM Tris-HCl and 150 mM NaCl) were loaded into a capillary cell using an automatic robotic sample changer [47]. All measurements were performed at 15°C. For each measurement, a total of 20 50-ms frames were recorded using a Pilatus 2M detector (Dectris, Switzerland). The Primus program [48] from the ATSAS package [49] was used for data processing, buffer subtraction and radius of gyration (R_g_) value calculation. All SAXS data were collected over the scattering vector s range from 0.0025 to 0.441 Å^-1^. The pair distance distribution function p(r) was calculated using GNOM [50].

### Modelling the low-resolution structure

The SAXS data were analysed using an ensemble optimization method (EOM) [51]. A pool of 10000 random conformers based on the MetC sequence was generated. A genetic algorithm was then applied to select an ensemble of models with the best fit to the experimental data [49]. The experimental SAXS data and MetC models from EOM have been deposited (SASBDB accession code: SASDBY5) at Small Angle Scattering Biological Data Bank (https://www.sasbdb.org) [52].

## Results

### *In silico* analysis suggests the unordered character of MetC

**Fig 1:**
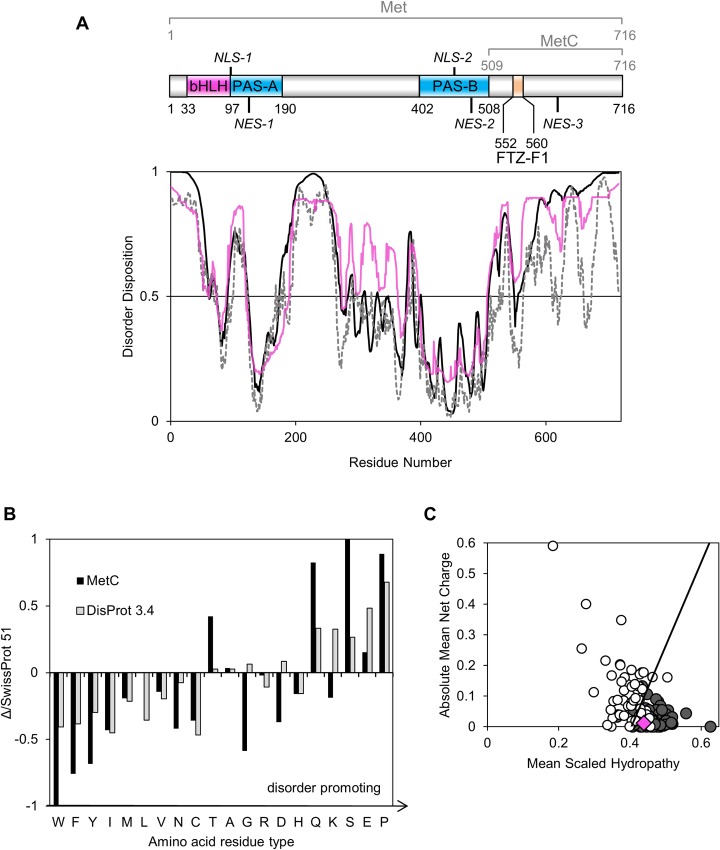
*In silico* analysis of the Met protein sequence. (A) The top panel represents the domain structure of Met [9,17,19]. Pink indicates the bHLH domain, whereas blue represents PAS domains. Orange signifies the interaction site for the FTZ-F1 factor. NLSs and NESs are marked [19,20]. The bottom panel presents a prediction of disorder regions based on the amino acid sequence of the Met protein. Calculations were performed using PONDR-VLXT (solid black line), IUPred (dashed black line) and GeneSilico MetaDisorder (solid pink line) software. A score over 0.5 indicates a high probability of disorder. (B) Amino acid composition. Composition Profiler [24,29] was used to analyse the amino acid composition. The results obtained for MetC (black bars) were combined with the amino acid distributions in proteins experimentally identified as IDPs (grey bars) [24]. All results refer to the SwissProt 51 database and the amino acid distributions in proteins in nature [53]. The amino acids are arranged in order of increasing disorder-promoting capacity. Values above zero indicate abundance, and values below zero indicate a deficit of a given residue. (C) Charge-hydropathy plot. The Uversky plot [30] compares the absolute, mean net charge and the mean hydropathy of disordered (open circles) and ordered proteins (grey circles). The boundary between ordered and disordered proteins is shown. The pink diamond corresponds to MetC.

To estimate the occurrence of putative IDRs in the structure of the *D*. *melanogaster* transcription factor Met, *in silico* analyses of its amino acid sequence were performed. The protein disorder predictors PONDR-VLS2 [31,32], PONDR-FIT [33], DISOPRED2 [34], FoldIndex [35] and IUPred [27] were used for Met sequence analysis. Since the results of all employed predictors were compatible, only two representative results are shown (Fig 1A). Additionally GeneSilico MetaDisorder server [36] was used to average the results (Fig 1A). All performed analyses indicate that Met is partially disordered, mainly in the C-terminal region (MetC). Indeed, only a short fragment of the MetC sequence is predicted to adopt a fixed 3D structure. In addition, the N-terminus (1–68 aa) and two short regions (99–123 and 192–265 aa) in the remaining part of Met exhibit an unordered character. These results are in agreement with a previous analysis described by Ashok *et al*. [9], indicating the occurrence of preserved domains characteristic for bHLH-PAS transcription factors family in Met sequence. Amino acid residues corresponding to the bHLH, PAS-A and PAS-B domains are primarily defined as ordered (Fig 1A). Further *in silico* analyses were performed on the MetC sequence (509–716 aa). Composition Profiler software was used to assess the abundance or depletion of the amino acid contents relative to the SwissProt 51 database, indicating the amino acid distributions in proteins found in nature [53]. The results for MetC were combined with the amino acid distributions in proteins experimentally determined to be IDPs and deposited in the DisProt 3.4 database [24]. Compared to the SwissProt 51 database, the MetC sequence is poor in nonpolar order-promoting residues (W, F, Y, I and N) and rich in disorder-promoting amino acids (Q, S and P) (Fig 1B) [54]. Interestingly, the MetC sequence, in comparison to the DisProt 3.4 database, is depleted in G and K residues defined as disorder promoting (Fig 1B). Furthermore, the amount of D and T residues is atypical for an IDP (Fig 1B); however, these amino acids are classified as disorder-neutral [54]. The depletion in D is favourable, since this residue could has an ordering effect, forming hydrogen bond to a backbone amide [55]. Summing up the amount of disorder-promoting amino acid residues is significant (S2 Fig) and indicates MetC disorder.

The above-mentioned analyses reveal that the amino acid composition of MetC is, to some extent, similar to those of proteins deposited in the DisProt 3.4 database [24] and may exhibit properties of IDPs. To test this hypothesis, an Uversky diagram was generated [56]. This diagram plots the mean net charge versus the mean hydrophobicity and distinguishes IDPs from ordered proteins. MetC occupies a rather ambiguous position near the boundary of the plot (Fig 1C), which is occupied by both ordered and disordered proteins.

The results of our *in silico* analyses were not sufficiently consistent, and thus, it was impossible to unambiguously define the structural characteristics of MetC. We therefore decided to perform an *in vitro* analysis to obtain insight into the MetC structure.

### Expression and purification of MetC

**Fig 2:**
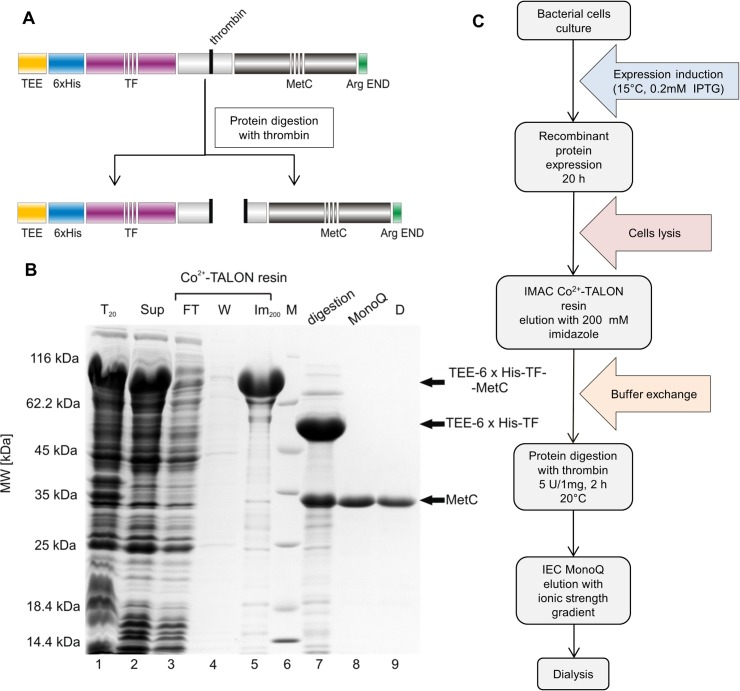
MetC purification. (A) A schematic illustration of a recombinant construct for MetC. The protein was N-terminally tagged with TEE-6×His-TF. After thrombin digestion, MetC was obtained. (B) Commassie Brilliant Blue R 250-stained SDS-PAGE analysis of the samples was performed following the expression and purification of MetC. Lane 1, the bacterial protein fraction; lane 2, the soluble protein fraction; lane 3, the fraction containing proteins not bound to the Co^2+^-TALON resin; lane 4, proteins washed from the Co^2+^-TALON resin with buffer B; lane 5, the combined fractions after elution with buffer C; lane 6, molecular mass standards; lane 7, protein after digestion with thrombin; lane 8, the combined MetC fractions after ion-exchange chromatography; lane 9, purified MetC after dialysis against buffer F. (C) A schematic of the MetC expression and purification process. For more details, see the Materials and Methods section.

To enable the *in vitro* analysis of the molecular properties of MetC, an expression and purification protocol was developed and optimized. Since expression from the pQE80L vector (QIAGEN) with a 6×His tag was not effective, and the protein was present mainly in the bacterial pellet (data not shown), we decided to use the pCold^TM^ TF DNA vector (Takara). The pCold^TM^ TF vector introduces a TEE, a 6×His tag and TF at the beginning of the protein sequence (Fig 2A). TF is the only ribosome-associated chaperone known to exist in bacteria [57] and is commonly used to enhance the stability and solubility of target proteins for proper folding. The results of SDS-PAGE (Fig 2B) indicate that the recombinant TEE-6×His-TF-MetC protein was successfully expressed (Fig 2B, Lane 1) and was present in the soluble fraction (Fig 2B, Lane 2). The MM of TEE-6×His-TF-MetC calculated with ProtParam was 74.7 kDa, whereas that determined based on electrophoretic mobility in SDS-PAGE was 77.9 ± 4.2 kDa. Thus, the size of the overexpressed protein corresponds to that of TEE-6×His-TF-MetC.

**Fig 3:**
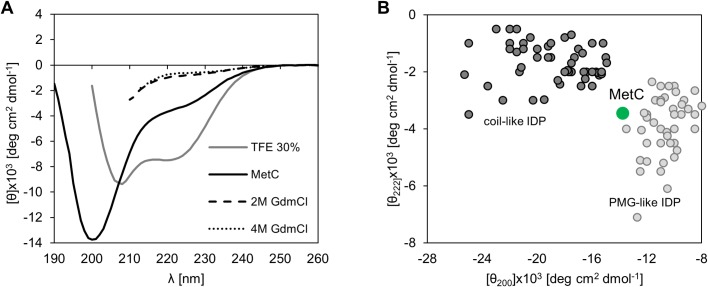
The far-UV CD spectra of MetC. (A) The CD spectra recorded in buffer F at 20°C for MetC in its native state (black solid line) in the presence of 30% TFE (grey solid line) and 2 M GdmCl (dashed line) or 4 M GdmCl (dotted line). (B) A double-wavelength plot showing [θ]_222_ versus [θ]_200_ for coil-like (dark grey) and PMG-like IDPs (light grey) [65]. The data set was obtained from a previously published work [64]. The green circle corresponds to MetC.

The efficient MetC-purification procedure required several steps (Fig 2C). The 6×His tag attached to MetC enabled us to use IMAC with a Co^2+^-TALON resin as the first step of purification (Fig 2B, Lane 5). The TEE-6×His-TF-MetC was eluted with 200 mM imidazole. The TEE-6×His-TF tag was removed by digestion with thrombin (2 h at 20°C), producing untagged MetC (Fig 2A and 2B, Lane 7). However, the separation of the digestion products was problematic because IMAC with Co^2+^-TALON resin and molecular gel filtration were unsuccessful. In both experiments, MetC and the TEE-6×His-TF tag were eluted together (data not shown), probably due to the fact, that TF remained complexed to the MetC. We therefore employed MonoQ anion exchange chromatography (Amersham Pharmacia Biotech). MetC was eluted with a NaCl gradient, what allowed the separation of MetC from the TEE-6×His-TF tag and other contaminants (Fig 2B, Lane 8). In the final step, the MetC sample was dialysed against buffer F (20 mM Tris-HCl and 150 mM NaCl) to obtain the protein in a buffer with a standard concentration of NaCl (Fig 3B, Lane 9). The expression and purification process (Fig 2C) typically yielded up to 2 mg of homogeneous MetC from 300 ml of culture medium.

To verify whether the obtained MetC sample had the correct MM, ESI mass spectrometry was used. The resulting MM value of 23 394.0 ± 15.6 Da differed from that calculated with the ProtParam tool (23 396.0 Da) by 0.01%.

Purified MetC appeared as a single band on the 12% SDS-PAGE gel (Fig 2B, Lane 9). Its electrophoretic mobility was much smaller than expected for a 23.40 kDa protein. The calculated MM of MetC based on its electrophoretic mobility was approximately 34.0 ± 2.0 kDa. This apparently higher MM in SDS-PAGE is a behaviour often observed for disordered proteins. Their unique amino acid composition results in reduced SDS binding and atypical mobility in SDS-PAGE experiments [25].

### Secondary structure content analysis by far-UV CD reveals that MetC is largely unstructured

CD spectroscopy is used to evaluate the secondary structure content and folding properties of proteins [58]. Different structural elements are represented by characteristic CD spectra in the far-UV region. Artificially created α-helical polypeptides present negative bands at 222 nm and 206 nm [59]; β-strand polypeptides result in negative bands at 218 nm [60]; and IDPs are characterized by a negative peak at approximately 200 nm and almost no peak at approximately 222 nm [25]. These markedly different features of the CD spectrum curve for each secondary structure make identifying partially or fully disordered proteins easy. We decided to use CD values with corresponding HT under 750 V (S1 Fig). Such selection allowed us to use high amount of values for deconvolution. Despite a greater possibility of generating errors, the results are still sufficiently reliable.

The CD spectrum of MetC exhibits properties typical for IDPs (Fig 3A). It shows a minimum near 200 nm (-13.7×10^−3^ deg·cm^2^·dmol^-1^), and a small negative peak is present near 222 nm (-3.5×10^−3^ deg·cm^2^·dmol^-1^), suggesting the existence of some residual ordered structure. The spectrum was analysed with CDPro spectra deconvolution software using the CONTIN/LL algorithm and the SDP48 base [41] representing a set of 48 proteins (43 soluble and 5 denatured). The results (Table 1) revealed that the MetC is mainly unordered (64.0 ± 5.3%) and that the dominant type of ordered structures is β-strands (23.3 ± 5.4%); α-helical structures are nearly absent.

**Table 1:** Determination of the structure of MetC by CD in the far-UV region.

Agent	α-Helix (%)			β-Strand (%)			Turns (%)	Unordered (%)
	Regular	Distorted	Total	Regular	Distorted	Total		
-	0. 1 ± 1.5	1.6 ± 1.7	1.7 ± 3.2	14.7 ± 3.4	8.6 ± 2.0	23.3 ± 5.4	11.0 ± 2.1	64.0 ± 5.3
30% TFE	17.8 ± 6.1	18.2 ± 0.9	36.0 ± 7.0	14.1 ± 3.0	12.9 ± 2.1	27.0 ± 5.1	12.7 ± 3.2	24.1 ± 1.0
2 M GdmCl	0.1 ± 1.2	1.6 ± 1.8	1.7 ± 3.0	7.1 ± 1.5	5.3 ± 1.1	12.4 ± 2.6	9.5 ± 3.3	76.5 ± 2.3
4 M GdmCl	0.2 ± 2.2	0.7 ± 2.1	0.9 ± 4.3	2.5 ± 2.1	2.1 ± 3.2	4.6 ± 5.3	2.7 ± 4.1	88.6 ± 3.4

CD spectra were analysed using CDPro spectra software. The CONTINLL algorithm on the SDP48 base was used to the deconvolution.

Changes in secondary structure content related to increasing concentrations of denaturing agent (i.e., GdmCl) provide significant information regarding the protein conformation and degree of protein compaction. We studied the impact of 2 M and 4 M GdmCl on MetC secondary structure content. At such concentrations, GdmCl absorbs so strongly that reliable CD data can only be recorded in a very narrow wavelength interval, even using cells of short path length (0.02 cm) [61]. From this reason, despite we recorded (Fig 3A) and analysed (Table 1) the data obtained after MetC incubation with GdmCl, our calculation results should be treated with a rough approximation. The addition of a denaturing agent resulted in less negative ellipticity value at approximately 222 nm (-0.7×10^−3^ deg·cm^2^·dmol^-1^), indicating the loss of residual structure (final amount of residual secondary structure was 14.1% and 5.5% for 2 M and 4 M GdmCl, respectively). This result proved that in the absence of GdmCl, MetC exhibits residual ordered secondary structure. After incubation with 4 M denaturing agent, the structure of MetC was almost fully disordered.

Uversky [62] demonstrated that certain chemical reagents (i.e., denaturants, osmolytes, binding partners, crowding agents, and counter ions) can affect IDP structure (i.e., more ordered structures can be observed). To determine how the conformation of MetC changes under various conditions, far UV-CD spectra were recorded. First, we examined the influence of 30% TFE, a known secondary structure stabilizer [63]. The result (Fig 3A) revealed that in the presence of TFE, negative peaks near 222 nm and 206 nm appear, suggesting that the amount of secondary structure increased. Indeed, MetC acquired α-helical structures (36.0 ± 7.0%, Table 1) at the expense of the disordered form (24.1 ± 1.0%, Table 1). The β-strand content remained stable (Table 1). The influence of 2 M and 4 M TMAO, which is known to be a gentle osmolyte that promotes the structuring of some proteins [64], was also analysed. The shape of the CD spectrum in the presence of 2 M TMAO was similar to that recorded in the absence of this reagent. The effect of a higher concentration of TMAO could not be analysed because MetC precipitated after a short incubation period (data not shown).

According to Uversky [65], IDPs can be divided into two groups: coil-like and PMG-like IDPs (Fig 3B). The differentiation is based on the ellipticity values at 200 and 222 nm, and each group is characterized by specific values. Referring to data obtained from the CD spectrum, MetC is located between two groups but falls closer to the area occupied by PMG-like IDPs (Fig 3B).

Altogether, the above results demonstrate the disordered character of the MetC structure. However, MetC has residual secondary structure that disappears under denaturing conditions. In addition, we hypothesize that MetC could have more ordered structure in some conditions.

### Hydrodynamic studies confirm the disordered character of MetC and the lack of a propensity for oligomerization

To verify the SDS-PAGE and CD results, which indicate the disordered character of the MetC structure, we decided to use additional independent techniques. SEC and AUC allowed us to gain insight into the shape-related hydrodynamic parameters of the protein [44,66].

**Fig 4:**
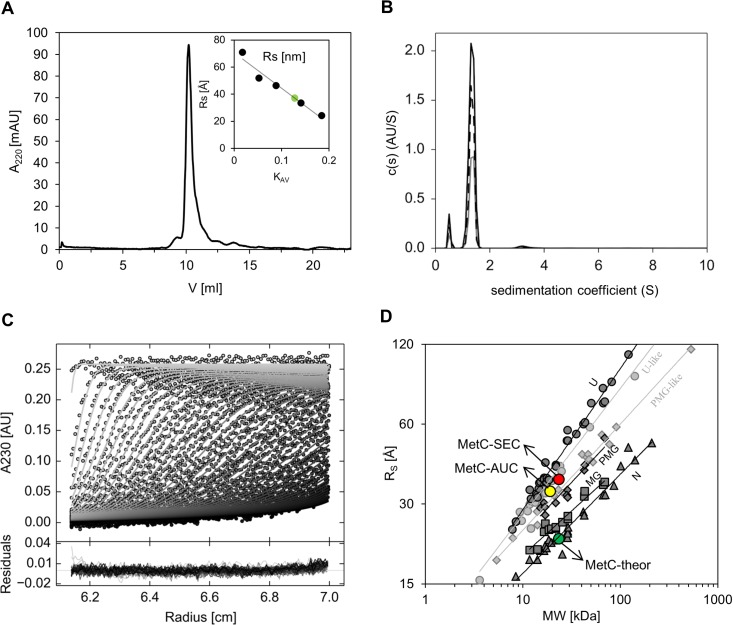
Hydrodynamic properties of MetC. (A) Analytical SEC. Experiments were performed on a Superdex75 10/300 GL column. The presented curve represents the elution volume of MetC. The inset shows the calibration curve determined using standard proteins (black dots). The green circle corresponds to MetC. (B) AUC analysis. The graph presents the sedimentation coefficient distributions c(s) derived via SEDFIT [44] from SV data for MetC at three different concentrations (0.30 mg/ml [black solid line], 0.17 mg/ml [black dashed line], and 0.08 mg/ml [grey solid line]) measured at 230 nm during the SV experiment at 50000 rpm at 20°C. (C) An example of the sedimentation profile of MetC. Representation of select experimental (circles) and fitted SV profiles (solid lines for MetC at 0.30 mg/ml). An rmsd value of 0.00674 confirms a good fit of the SV data. (D) The relationship between the hydrodynamic radii (R_S_) and the relative MMs of four equilibrium states for globular proteins (dark grey lines) and IDPs (light grey lines). The globular proteins are shown as dark grey symbols: native proteins (N, tringles), molten globules (MG, squares), pre-molten globules (PMG, diamonds), and 6 M GdmCl-unfolded proteins (U, circles). The IDPs are shown as bright grey symbols: U-like (circles), and PMG-like (diamonds). The data for globular proteins correspond to previously published work [68], and those for IDPs were published by Uversky [65]. Values for MetC are shown as circles: theoretical (green), experimentally determined with SEC (red) and experimentally determined with AUC (yellow).

In the SEC experiment, MetC was eluted as a single peak with an elution volume corresponding to an R_S_ of 36.0 ± 2.0 Å (Fig 4A, Table 2). This value was independent of protein concentration (data not shown) and was approximately 1.6 times greater than the 22.7 Å value calculated with assumption that MetC had a globular structure and a 23.40 kDa MM, from the equation log(R_S_) = -(0.204±0.023)+(0.357±0.005)log(MM) (Table 2). The experimentally determined volume (195.4 Å^3^) of the protein was much larger than the theoretical volume (49.0 Å^3^), whereas the density (0.12 kDa/ Å^3^) was much lower than the theoretical density (0.48 kDa/Å^3^) (Table 2). Thus, it can be concluded that the MetC molecule has a highly elongated structure or can form oligomers under experimental conditions. In addition, the MM determined via SEC (74.6 ± 3.0 kDa, 3-fold higher than theoretical 23.40 kDa) suggested that MetC could exist as an oligomer.

**Table 2:** Characterisation of MetC by SEC.

MM [kDa]	Rs [Å]		V_S_·10^3^ [Å^3^]		p·10^3^ [kDa/Å^3^]	
	Theor	Exp	Theor^a^	Exp^b^	Theor^a^	Exp^b^
23.40	22.7	36.0 ± 2.0	49.0	195.4	0.48	0.12

^a^Calculated using the theoretical R_S_.

^b^Calculated using the experimental R_S_.

To determine whether MetC is an oligomer or an extended monomer, AUC experiments were performed for three concentrations of MetC (Fig 4B and 4C). The determined root-mean-square deviation (rmsd) values were low and proved a very good fit for the obtained results. The values of the sedimentation coefficient (S_20,w_) were concentration independent, and the experimentally determined R_S_ values (ca 33 Å) (Table 3) were in good agreement with the SEC data (36.0 ± 2.0 Å). The frictional ratios f/f_0_ help to verify the character of the protein. For globular compact proteins, f/f_0_ values of approximately 1.2–1.25 are typical. In addition, the increase of f/f_0_ with MM is very slight (from 1.19 to 1.25 for MMs between 20 and 200 kDa). In the case of IDPs, the f/f_0_ ratio is much higher, and the f/f_0_ increases only slightly with the MM. For example, f/f_0_ is typically 2.1 for a 20 kDa protein, 3.0 for a 200 kDa coil-like IDP, 1.75 for a 20 kDa and 2.05 for a 200 kDa PMG-like IDP [66]. The frictional ratios f/f_0_ were calculated for MetC from the relation between the size and shape of the molecule [44,67]. Results over 1.8 (Table 3) indicated that the protein had an elongated shape. AUC can also be applied for MM determination. In our case, the MM of MetC determined via AUC (Table 3) was smaller than that determined via ESI mass spectrometry (19 kDa versus 23.40 kDa). Such a discrepancy is often observed for IDPs because one must account for a heterogeneous population of macromolecules characterized by slightly different s-values, which can result in boundary spreading. Thus, the application for calculating an average s-value and the apparent diffusion coefficient will lead to an apparent molecular mass [68]. Nevertheless, our AUC results proved that MetC exists in buffer as an extended monomer and does not exhibit a propensity for oligomerization.

**Table 3:** Characterisation of MetC by sedimentation velocity AUC.

				Main peak			
Concentration [mg/ml]	rmsd	f/f_0_	s_20,w_ (S)	s (S)	%	Rs [Å]	MM [kDa]
0.08	0.00457	1.88	1.392	1.342	88.9	33.2	19.0
0.17	0.00582	1.84	1.382	1.332	89.7	32.0	18.2
0.3	0.00663	1.91	1.383	1.33	90.5	33.8	19.2

Globular proteins can be assigned to four equilibrium conformational states that are characterized by differences in the dependence of the R_S_ on the relative MM: native proteins (N), molten globules (MGs), pre-molten globules (PMGs) and 6-M GdmCl-unfolded proteins (U). Two additional IDP states also exist: U-like and PMG-like subclasses (Fig 4D) [64,69]. The much larger R_S,exp_ determined by SEC and AUC relative to the calculated value placed MetC in the area occupied by IDPs on the plot relating R_S_ and MM (Fig 4D). Thus, MetC clearly exhibits properties of an IDP. According to equations derived by Tcherkasskaya *et al*. [69] correlating the MM and the R_S_ for different conformational states of protein, we obtained for MetC (23.4 kDa) 33.6±0.4 Å for PMG-like state and 38.7±0.4 Å for U-like state. Our experimental results (36 Å with SEC and 33 Å with AUC) can suggest that MetC state closer to PMG-like IDP.

Based on the hydrodynamic properties determined by SEC and AUC we can conclude that MetC exists in solution as a highly disordered molecule. It has an elongated shape and has no propensity to create oligomers in solution.

### SAXS studies and modelling of the low-resolution MetC structure reveal its extended shape

SAXS is a method, which is complementary to crystallography and NMR, and is widely used for studying the low-resolution structure of macromolecules in solution [70,71]. This method can provide insight into tertiary structure of biological macromolecules and enable researchers to distinguish between ordered and disordered proteins [70,71]. The scattering profile of IDPs is characteristic [71]; thus, SAXS was employed to confirm the SEC and AUC results and to obtain additional information regarding the structure of MetC.

**Fig 5:**
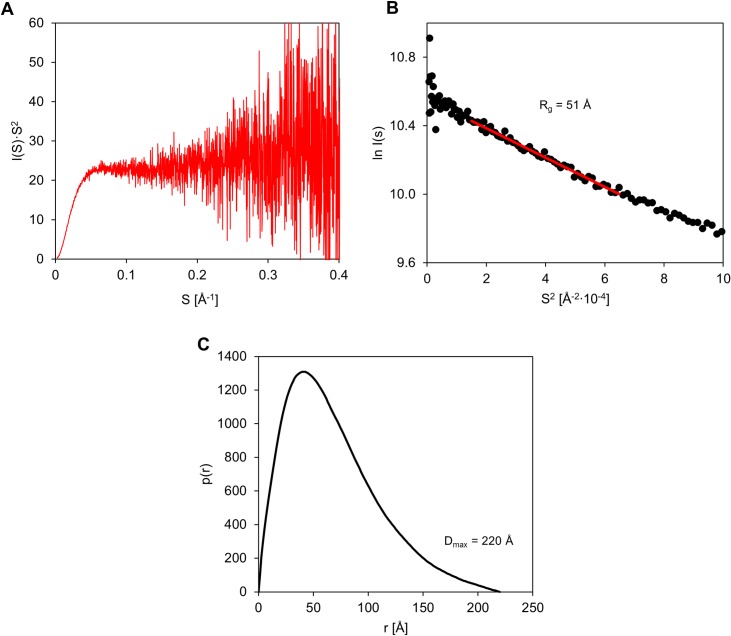
SAXS characteristics of MetC. (A) Kratky plot analysis. The intensity of scattering is plotted as I(s)·s^2^ versus s. The plot has no maximum, and at higher s values, it plateaus. (B) Gunier plot of SAXS data. The experimental points exhibit good fit to Gunier equation, which indicates also good monodispersity of the MetC in solution. (C) The pair distribution function p(r) of MetC. The asymmetric shape of the function indicates an elongated protein shape.

The Kratky plot is a representation of scattering intensity [I(s)·s^2^] as a function of the vector s [72], and is used for a qualitative analysis of the protein scattering profile. The profile of the Kratky plot for MetC is presented in Fig 5A. The SAXS curve does not have a clear maximum, and at higher values of scattering vector s reaches a plateau that is characteristic of IDPs. However the shape of the curve can suggest that the MetC is not fully disordered and could have some residual structure. The radius of gyration characterising MetC in solution was calculated from linear part of Gunier plot [73] in the range 0.014< s^2^< 0.064 nm^-2^ (corresponding to sR_g_ limits: 0.611–1.285) (Fig 5B). The SAXS data presented in Gunier plot show linear character (Fig 5B), also in low-s region, which is a good indicator of the monodispersity of MetC in solution. R_g_ for MetC, estimated from Gunier plot, was 51 Å. We did not observe any concentration dependence effect in the SAXS curves (data not shown).

The pair distance distribution function p(r) represents the distribution of all interatomic distances within the molecule and provides information about the size and shape of the molecule [74]. The p(r) function, characterising MetC molecule, was calculated using GNOM and experimental SAXS data in the s-range: from 0.006 to 0.441 Å^-1^. The p(r) plot reveals two main structural parameters: the radius of gyration (R_g_) (determined independently from R_g_ estimation using the Guinier equation) and the maximal intramolecular distance (D_max_) within the molecule [75]. The p(r) function for MetC possesses an asymmetric shape (Fig 5C). The R_g_ value, determined on the p(r) function using experimental SAXS data (the s-range: from 0.006 to 0.441 Å^-1^), was 56 Å and was similar to R_g_ calculated from Gunier plot. The D_max_ value was 220 Å. The theoretical R_g_ for MetC was calculated using Flory’s equation, relating it to the number of amino acid residues (N) in the unstructured (disordered) protein (Eq 3) [76].

$$R_{g}=(2.54\pm 0.01)∙N^{\left(0.522\pm 0.01\right)}$$ (3)

Comparison between the experimental and the theoretical R_g_ value expected for an IDP allows drawing meaningful conclusions about the conformational state of the protein under study. The value 42.22 Å, derived from Flory’s equation for MetC, is a value even lower than the value derived from SAXS experiments (over 50 Å). Additionally the ratio between experimentally determined R_g_ and R_S_ (33 Å, as determined with AUC) was calculated. This parameter is useful to determine the shape of the molecule in solution. The theoretical value of R_g_/R_S_ ratio is 0.778 for a hard sphere, from 0.875 to 0.987 for oblate ellipsoids, and from 1.36 to 2.24 for prolate ellipsoids. The value obtained for MetC was 1.62 and indicate highly elongated shape of MetC molecule [77].

These results indicate that MetC has a behaviour typical of IDPs adopting an extended conformation with a low density and large size compared to globular molecules, and confirm the disordered character of MetC.

**Fig 6:**
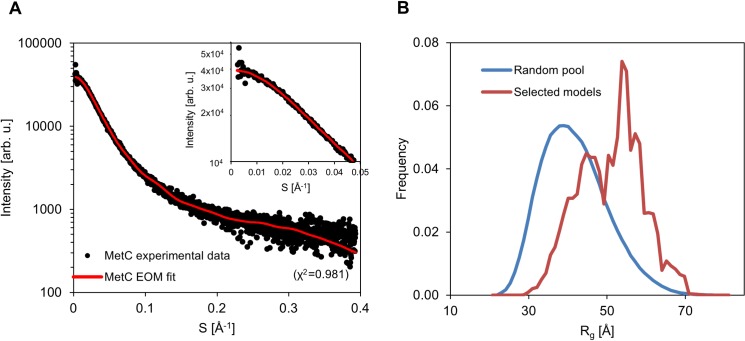
Modelling of MetC low resolution structure in solution. (A) The fit between the experimental scattering profile (black dots) and the profile back-calculated from the selected sub-ensemble (generated with EOM) (red) indicates a good match (χ^2^  = 0.981), inset–the zoom of low-s region. (B) Radii of gyration profile of the initial random pool of MetC structures (blue) and profile of the final conformational sub-ensemble (red).

**Fig 7:**
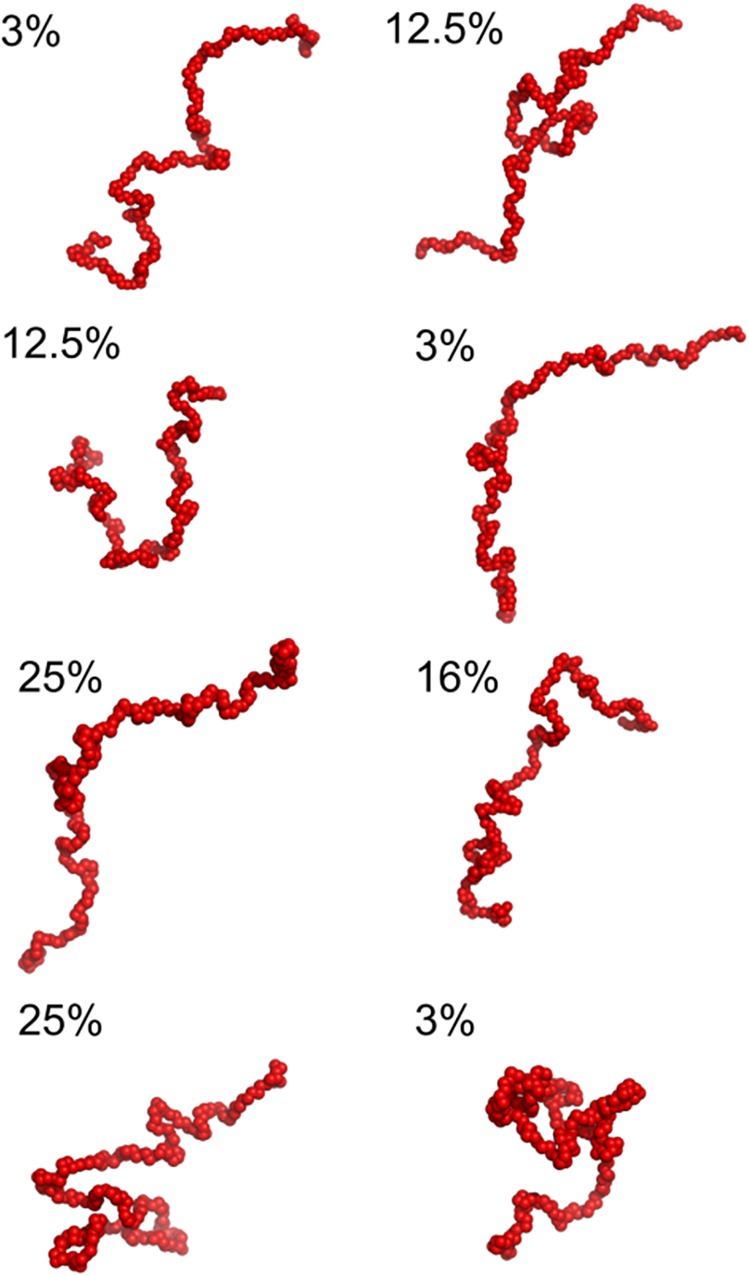
Flexibility of MetC in solution. Population of MetC low resolution models generated on the basis of SAXS data and using EOM modelling. The selected models are presented with the percentage contribution, estimated from final population of EOM models.

In order to achieve additional insights into the conformational properties of MetC, we used EOM. EOM first generates a pool of 10000 random coil conformers and then selects a sub-ensemble that best fit the scattering profile using a genetic algorithm. The fit between the experimental scattering profile and the profile back-calculated from the selected sub-ensemble is very good (χ^2^ of 0.981) (Fig 6A). The R_g_ distribution of the final conformational sub-ensemble (Fig 6B) is bimodal and slightly shifted towards more extended conformations as compared to the initial random pool. Fig 7 shows the SAXS-derived conformers of the final conformational ensemble along with their relative frequency. Essentially, they can be grouped into two main groups—models with an open and extended conformation and models containing a small "locally entangled" domain (Fig 7). The latter group probably reflects the presence of a small structured region within the MetC chain. Altogether, the results of the SAXS analysis confirm the disordered character of MetC and its asymmetric elongated shape.

## Discussion

According to sequence alignment and other *in silico* analyses, Met was assigned to a family of transcription factors with structurally preserved and crucial bHLH and PAS domains [9]. These domains are responsible for DNA and ligand (JH) binding, homodimerization, and interactions with proteins, such as Gce [78], steroid receptor co-activator (SRC) [79,80] and Hsp 90 [81]. However, the sequence of the MetC region is has no homology to any sequence deposited in the PDB (available at http://www.rcsb.org/pdb/home/home.do). In this study, we show that MetC is an IDR with a possibility of folding.

It has been shown the C-termini of bHLH-PAS transcription factors play a crucial role in regulating the activity of these proteins [12]. Uversky [82] has reported that intrinsically disordered termini of proteins are able to perform a number of rather unusual *functional tricks* in their natural states. He suggested that for many proteins, the most important region is the intrinsically disordered tail. The C-terminal region of Met interacts with FTZ-F1 through its LIXXLL (552-560aa) and Q_R_ (630-647aa) sequences [17,83–84]. This NR has been shown to bind the *Drosophila* Ftz protein and to influence its DNA binding specificity [18]. The SAXS and denaturing analyses performed here suggest that MetC is not fully disordered and that it contains some residual structures (Figs 3A and 7, Table 1). That may correspond to the presence of a short helical region formed by the LIXXLL sequence and interacting with FTZ-F1. Interestingly, in its interaction with Met, FTZ-F1 exploits not the canonical charge clamp but hydrophobic residues of its ligand-dependent activation function 2 (AF-2) domain [17,85]. Interaction with FTZ-F1 is possible only in the presence of JH [83]. We suggest that JH binding can affect the conformation of MetC by changing of PAS-B conformation and the following propagation of this change to intrinsically disordered MetC, forcing it to adopt a more fixed structure, thereby enabling FTZ-F1 to bind. It is possible even when JH is bound by the PAS-B domain rather than MetC [3]. FTZ-F1 has been shown to be an exclusively nuclear protein [86,87], whereas Met is a shuttling protein containing two NLSs and three NESs that regulate its transport between the nucleus and the cytoplasm. One of these NESs has been reported to be located in the MetC fragment [19,20]. He *et al*. [81] showed that Met is primarily localized in the cytoplasm in larval fat body cells when the amount of JH is low. They proposed that prior to JH binding, Met remains in an inactive conformation that is weakly associated with Hsp83. The appearance of JH stimulates the Met-Hsp83 interaction by inducing a conformational change. We suggest that changes in the JH-binding PAS-B domain influence the structure of the neighbouring MetC region. Changing the conformation of MetC would influence its interaction with partner proteins and switch on the NES localized in this area, resulting in the nuclear transport and activation of Met.

Post-translational modifications (PTMs) are involved in targeting proteins to specific subcellular compartments, binding ligands or partner proteins, and regulating the functional states of proteins involved in signal transduction pathways [83]. We analysed *in silico* the possibility of MetC phosphorylation (data not shown) and found over 20 amino acid residues (serine and threonine) characterized by a very high probability of PTMs that could affect the conformation of this region. It has been shown that the phosphorylation of N- and C-terminal disordered regions regulates the activity of Neurogenin2 (Ngn2), a bHLH transcription factor responsible for controlling neuronal differentiation [88]. In the case of p53, PTMs play an important role in regulating its binding affinity for various cellular partners [89]. Increasing the number of phosphorylation events on the intrinsically disordered N-terminal transactivation domain of p53 directly increases its affinity for the TAZ1, TAZ2 and KIX domains of CBP/p300 [90], whereas the intrinsically disordered C-terminal region is involved in downregulating DNA binding by the DNA binding domain [89,91].

An intriguing feature of IDPs is their ability to undergo a disorder-to-order transition upon functioning [65]. The experimentally determined MetC structural disorder with possibility of presence of some residual structures confirm its probability for folding and undergoing conformational changes for interactions with other proteins [65]. We used the experimental scattering curves of MetC for modelling of low-resolution models with EOM. Obtained results allowed us to propose a set of structures that best fit the SAXS data. Detailed analysis of the resulting pool of models made it possible to determine the percentage shares of individual flexible MetC models. Specific MetC structures contributed to the final pool of EOM models from 3 to 25%. Essentially, they can be grouped into two main groups—models of the open and extended conformation and models containing small "locally entangled" domain (Fig 7). The latter group probably represents the conformations containing small structured fragment of the MetC chain. Using the population of MetC models obtained by EOM On the basis of SAXS modelling we can presume, that the extended shape and long unfolded regions lend MetC high flexibility and a dynamic structure. The multiplicity of conformation that MetC can adopt is crucial for effective activity of Met as a biological switch at the intersection of different signalling pathways in insects. All changes in its structure can result in important alterations in conformation, thereby affecting the activity or localization of the protein.

## Conclusion

In this paper, we present the results of a series of *in silico* and *in vitro* analyses of the structural properties of the *D*. *melanogaster* MetC. We demonstrated that MetC exhibits a highly disordered character with a possibility of folding and can undergo conformational changes. Specific MetC structures in the final pool of EOM models averaged from 3 to 25%. In addition, MetC is not fully disordered. Possible secondary structures could be responsible for FTZ-F1 recognition. MetC exists in solution in extended and flexible forms with no propensity for oligomerization. It is predicted as presenting a high predisposition for post-translational phosphorylation. We hypothesize that the intrinsic disorder of MetC enables its interaction with multiple unknown partners and is responsible for its function, not only as a factor mediating JH signal transduction but also in connecting the JH and 20E signalling pathways during the development and maturation of *D*. *melanogaster*.

## Supporting Information

S1 Fig High tension (HT) function for CD spectra. HT functions for CD spectra recorded in buffer F at 20°C for MetC in its native state (black solid line) in the presence of 30% TFE (black dashed line) and 2 M GdmCl (grey solid line) or 4 M GdmCl (grey dashed line).
(TIF)

S2 Fig Amino acid sequence of MetC. Amino acid sequence of MetC. All disorder-promoting amino acids are highlighted in yellow.
(TIF)

S1 Table CD data. CD data for MetC in native state and after 1 h incubation with 30% TFE, 2M GdmCl or 4M GdmCl. All values are presented in the molar residual ellipticity units.
(PDF)
