# Supplementary figures and images for: Intrinsic Disorder of the C-Terminal Domain of Drosophila Methoprene-Tolerant Protein
Source: PLoS One. 2016 Sep 22;11(9):e0162950. doi: 10.1371/journal.pone.0162950 (PMC5033490; doi:10.1371/journal.pone.0162950)

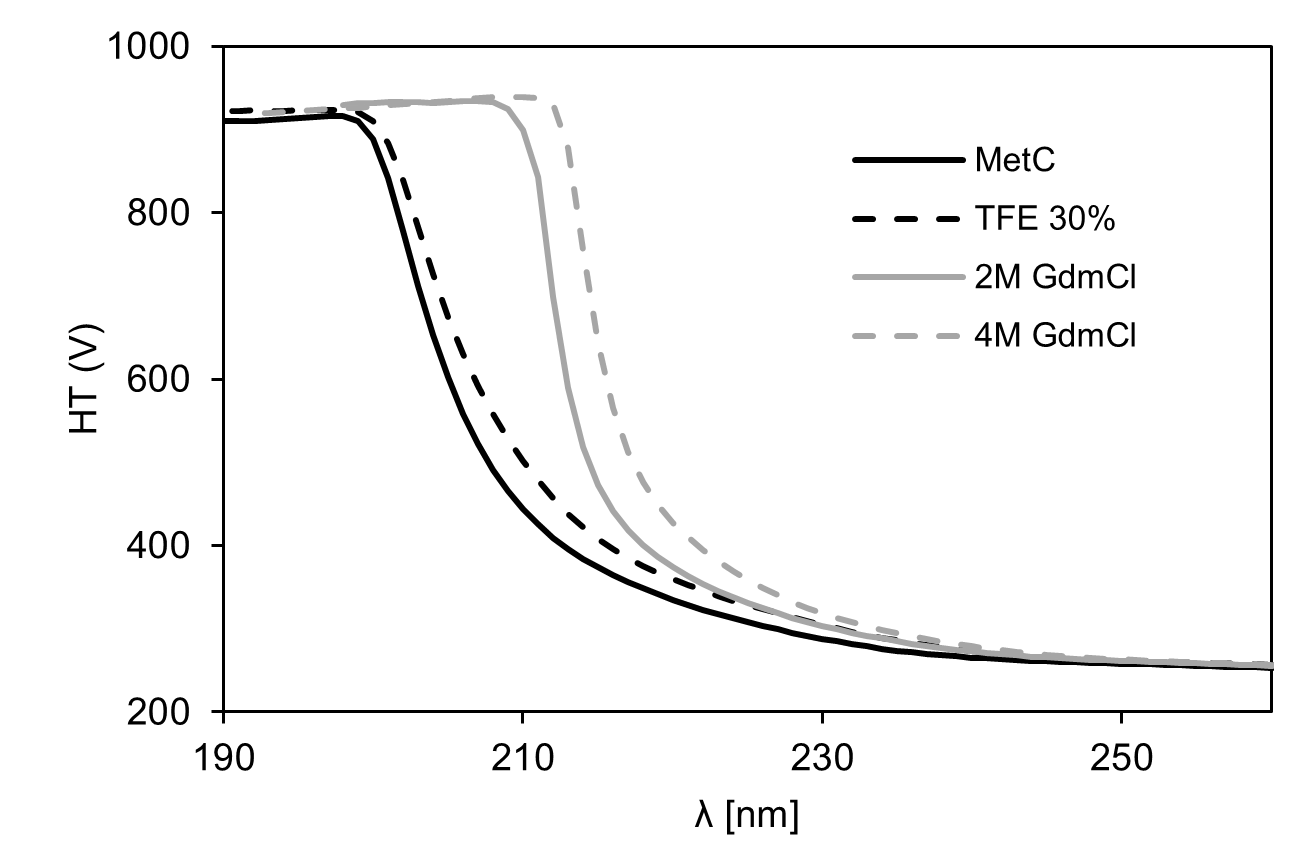

Supplement: S1 Fig — HT functions for CD spectra recorded in buffer F at 20°C for MetC in its native state (black solid line) in the presence of 30% TFE (black dashed line) and 2 M GdmCl (grey solid line) or 4 M GdmCl (grey dashed line). (TIF) [file pone.0162950.s001.tif]

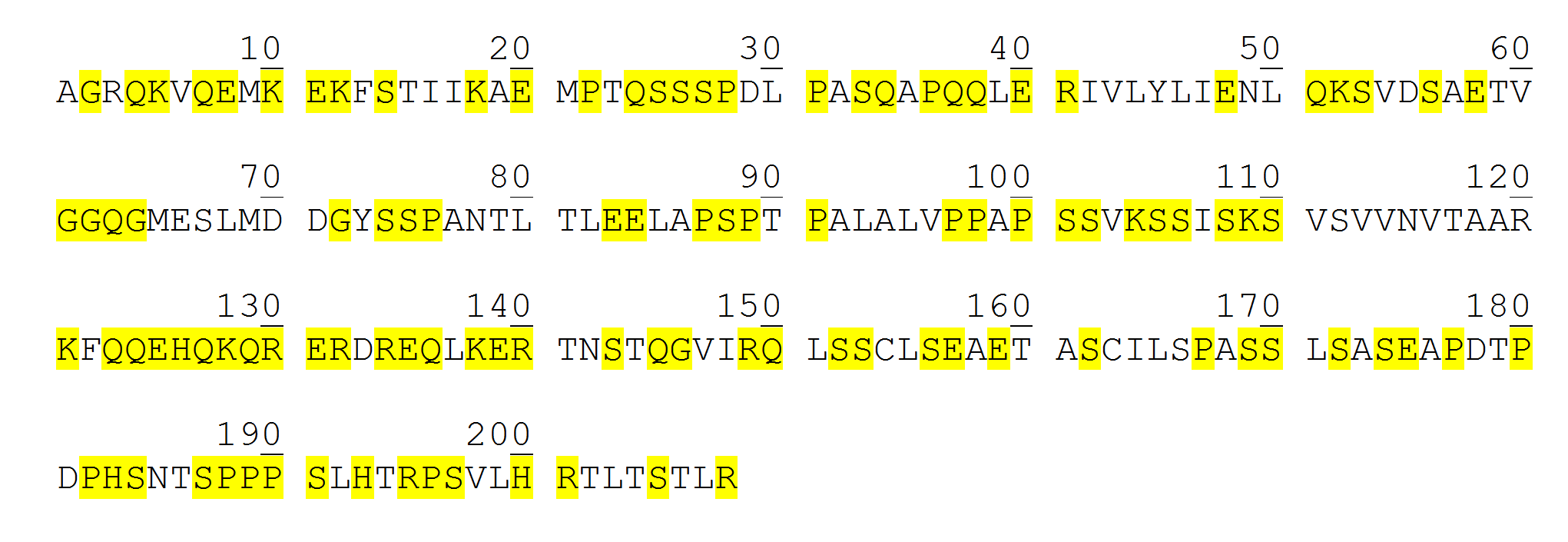

Supplement: S2 Fig — Amino acid sequence of MetC. All disorder-promoting amino acids are highlighted in yellow. (TIF) [file pone.0162950.s002.tif]
