# Supplementary material for: Intrinsic Disorder of the C-Terminal Domain of Drosophila Methoprene-Tolerant Protein
Source: PLoS One. 2016 Sep 22;11(9):e0162950. doi: 10.1371/journal.pone.0162950 (PMC5033490; doi:10.1371/journal.pone.0162950)
Supplement: S1 Table — CD data for MetC in native state and after 1 h incubation with 30% TFE, 2M GdmCl or 4M GdmCl. All values are presented in the molar residual ellipticity units. (PDF) [file pone.0162950.s003.pdf]

	MetC [molar residual ellipticity]					MetC [molar residual ellipticity]			
Wavelenght [nm]	-	30% TFE	2M GdmCl	4M GdmCl	Wavelenght [nm]	-	30% TFE	2M GdmCl	4M GdmCl
260	-28.00	0.00	0.00	0.00	224	-3297.13	-7139.19	-849.66	-638.01
259	-8.80	6.93	0.81	-8.32	223	-3372.42	-7297.50	-873.20	-623.77
258	20.49	1.33	-1.66	-0.42	222	-3463.55	-7406.91	-889.30	-677.65
257	21.77	3.90	-4.95	1.34	221	-3568.04	-7483.19	-923.84	-705.22
256	-10.85	8.52	-10.62	-9.45	220	-3676.21	-7503.56	-982.22	-734.56
255	-19.36	3.66	-9.06	-12.24	219	-3790.89	-7485.60	-1048.20	-836.56
254	-25.37	-0.14	-17.47	-14.03	218	-3898.50	-7456.36	-1083.47	-924.20
253	-16.70	-10.80	-11.61	-19.66	217	-4067.09	-7457.84	-1224.26	-1080.23
252	-5.14	-8.46	-13.10	-14.42	216	-4246.09	-7450.54	-1348.13	-1194.75
251	-16.15	-24.37	-15.68	-25.06	215	-4480.18	-7502.48	-1512.45	-1374.96
250	-44.54	-35.53	-22.74	-28.18	214	-4755.99	-7564.83	-1677.12	-1668.94
249	-61.72	-52.66	-37.42	-45.54	213	-5131.74	-7731.58	-1893.37	-1789.81
248	-96.62	-75.63	-50.58	-52.10	212	-5587.61	-8006.57	-2141.84	-1877.97
247	-122.07	-118.28	-54.24	-58.94	211	-6206.54	-8326.29	-2525.56	-1072.00
246	-163.23	-164.15	-68.73	-80.18	210	-6912.72	-8702.18	-2710.12	559.00
245	-226.94	-223.13	-85.94	-99.15	209	-7722.84	-9131.32	-2101.26	-560.28
244	-292.72	-293.02	-119.97	-117.54	208	-8562.45	-9374.54	-1274.78	343.87
243	-369.58	-391.44	-138.11	-142.76	207	-9442.17	-9260.23	-380.25	497.47
242	-436.80	-518.03	-164.96	-172.69	206	-10338.00	-9091.37	-1113.50	-284.63
241	-520.74	-668.04	-207.62	-212.79	205	-11265.70	-8456.72	-1828.06	1605.50
240	-616.07	-839.63	-232.71	-236.61	204	-12100.20	-7546.84	-1696.18	1458.51
239	-739.02	-1071.75	-268.54	-274.27	203	-12881.4	-6491.79	1844.41	-930.07
238	-858.93	-1326.53	-307.00	-302.21	202	-13360.40	-5018.23	1276.62	1096.14
237	-999.63	-1627.16	-352.21	-347.12	201	-13706.70	-3489.30	-540.46	-2288.59
236	-1132.57	-1984.58	-402.13	-397.03	200	-13747.20	-1615.06	448.47	-1170.79
235	-1324.28	-2379.08	-460.51	-421.57	199	-13629.20	86.42	687.26	16.93
234	-1535.36	-2810.39	-511.88	-463.11	198	-13060.50	497.73	-54.23	-55.53
233	-1748.67	-3283.98	-551.00	-488.41	197	-12235.20	486.32	-2198.15	-2690.94
232	-1943.71	-3784.70	-585.99	-516.14	196	-10740.10	1090.76	-599.40	1895.73
231	-2152.41	-4303.60	-627.92	-538.60	195	-9307.43	-317.97	1873.12	1428.90
230	-2353.11	-4828.91	-669.74	-560.74	194	-6944.71	-796.68	-1786.00	-650.81
229	-2548.16	-5334.90	-718.00	-582.77	193	-5681.62	493.83	-500.16	463.56
228	-2739.90	-5815.56	-723.25	-566.26	192	-4074.50	3092.51	-903.85	620.49
227	-2911.85	-6217.11	-760.63	-579.73	191	-2670.64	2112.39	-1843.20	-2208.98
226	-3056.16	-6610.26	-793.06	-619.47	190	-1451.76	-147.40	-22.65	98.04
225	-3191.74	-6894.79	-803.59	-617.09					
